# Radiobiological model-based approach to determine the potential of dose-escalated robust intensity-modulated proton radiotherapy in reducing gastrointestinal toxicity in the treatment of locally advanced unresectable pancreatic cancer of the head

**DOI:** 10.1186/s13014-020-01592-6

**Published:** 2020-06-22

**Authors:** Vijay P. Raturi, Hidehiro Hojo, Kenji Hotta, Hiromi Baba, Ryo Takahashi, Toshiya Rachi, Naoki Nakamura, Sadamoto Zenda, Atsushi Motegi, Hidenobu Tachibana, Takaki Ariji, Kana Motegi, Masaki Nakamura, Masayuki Okumura, Yasuhiro Hirano, Tetsuo Akimoto

**Affiliations:** 1 Division of Radiation Oncology and Particle therapy, National Cancer Center Hospital East, 6-5-1 chome, Kashiwanoha, Kashiwa-shi, Chiba-ken 277-8577 Japan; 2 Course of Advanced Clinical Research of Cancer, Graduate school of Medicine, Juntendo University, Tokyo, Japan

**Keywords:** Pancreatic cancer, Intensity-modulated radiotherapy, Intensity-modulated proton therapy, Normal tissue complication probability

## Abstract

**Background:**

The purpose of this study was to determine the potential of escalated dose radiation (EDR) robust intensity-modulated proton radiotherapy (ro-IMPT) in reducing GI toxicity risk in locally advanced unresectable pancreatic cancer (LAUPC) of the head in term of normal tissue complication probability (NTCP) predictive model.

**Methods:**

For 9 patients, intensity-modulated radiotherapy (IMRT) was compared with ro-IMPT. For all plans, the prescription dose was 59.4GyE (Gray equivalent) in 33 fractions with an equivalent organ at risk (OAR) constraints. Physical dose distribution was evaluated. GI toxicity risk for different endpoints was estimated using published NTCP Lyman Kutcher Burman (LKB) models for stomach, duodenum, small bowel, and combine stomach and duodenum (Stoduo). A Wilcoxon signed-rank test was used for dosimetry parameters and NTCP values comparison.

**Result:**

The dosimetric results have shown that, with similar target coverage, ro-IMPT achieves a significant dose-volume reduction in the stomach, small bowel, and stoduo in low to high dose range in comparison to IMRT. NTCP evaluation for the endpoint gastric bleeding of stomach (10.55% vs. 13.97%, *P* = 0.007), duodenum (1.87% vs. 5.02%, *P* = 0.004), and stoduo (5.67% vs. 7.81%, *P* = 0.008) suggest reduced toxicity by ro-IMPT compared to IMRT. ∆NTCP _IMRT – ro-IMPT_ (using parameter from Pan et al. for gastric bleed) of ≥5 to < 10% was seen in 3 patients (33%) for stomach and 2 patients (22%) for stoduo. An overall GI toxicity relative risk (NTCP_ro-IMPT_/NTCP_IMRT_) reduction was noted (0.16–0.81) for all GI-OARs except for duodenum (> 1) with endpoint grade ≥ 3 GI toxicity (using parameters from Holyoake et al.).

**Conclusion:**

With similar target coverage and better conformity, ro-IMPT has the potential to substantially reduce the risk of GI toxicity compared to IMRT in EDR of LAUPC of the head. This result needs to be further evaluated in future clinical studies.

## Background

Pancreatic cancer is the fourth primary cause of cancer-related death in Japan [1]. Locally advanced unresectable pancreatic cancer (LAUPC) has a 5-year survival of < 5% [2]. The main treatment option for LAUPC is chemotherapy, while definitive chemoradiotherapy (CRT) plays a pivotal role in therapeutic management to enhance survival and quality of life [3].

Despite the use of modern radiotherapy (RT) delivery techniques, potentially gastrointestinal (GI), severe toxicity had been noted [4, 5]. Studies using escalated dose radiation (EDR) intensity-modulated radiotherapy (IMRT), PTV-based double scatter (DS), and pencil beam scanning (PBS) proton beam therapy (PBT) have reported improved local control and survival with the main limiting GI toxicity [6–8]. A study by Kelly et al. and Ben-Josef et al., in LAUPC using EDR-IMRT have reported ≥ grade 2 GI toxicity in approximately 15 to 20% patients [6, 9]. The study by Takatori et al., using hypo-fractionated concurrent gemcitabine PBT (GPT) for LAUPC 67.5 Gray equivalent (GyE) in 25 fractions) have reported 49.4% rate of gastric/duodenal ulcer [7]. The study by Terashima et al. treated 45 patients with hypo-fractionated GPT have reported 10% of grade ≥ 3 late gastric ulcer and hemorrhage [8]. However, studies using conventional fractionated 1.8 GyE/fraction concurrent PBT with a dosage of 59.4GyE for LAUPC have resulted in a modest decrease in GI toxicity with no grade 3 toxicity during treatment, or during follow-up. These studies had a limitation of small sample size and short median follow up [10, 11].

The clinical target volume (CTV) to PTV set-up margin (SM) alone cannot guarantee the adequate dose coverage of the CTV in PTV-based DS, PBS, and intensity-modulated proton therapy (IMPT) plans [12, 13]. In PTV-based IMPT plans, under- or over dosage inside the PTV can occur in the patient from deviation in the position of high in-field dose gradients from spot to spot due to set-up errors or range uncertainties. Hence, the comparison of PTV-based proton and photon treatment is certainly not precise [14]. The robust IMPT (ro-IMPT) plan can result in even dose gradients per field across the target volume and can reduce the risk of pencil beams ceasing directly in front of an adjoining normal tissue [13]. However, for pancreatic cancer, a dosimetric and radiobiological model-based comparative treatment planning study between ro-IMPT and IMRT has not yet been reported.

For the pancreatic head cancer surrounded circumferentially by gastrointestinal OARs (GI-OARs), IMRT was stated to be superior compared to DS proton therapy [15]. The dosimetric study by Thompson et al., in pancreatic head cancer reported no dosimetric evidence that DS and PBS proton therapy facilitates EDR more readily in comparison to IMRT, as surrounding GI-OARs receive incrementally higher doses using DS and PBS proton therapy [16]. The ro-IMPT with a spot-scanning technique would offer a more-fair comparison with IMRT plans. In-silico study by Stefaowicz et al., using EDR ro-IMPT in advanced pancreatic cancer, have reported a better target homogenous dose distribution and minimized dose to the OARs with a 3 beam design configuration with at least one non-coplanar beam [14].

The dose-volume analysis study is usually restricted to just certain specific DVH parameters that might not always correspond directly to a clinical outcome. The radiobiological normal tissue complication probability (NTCP) model using parameters emanate from reported toxicity rates in clinical trials, and it assesses the treatment plans by analyzing the information from the entire DVH. However, each toxicity endpoint has a specific NTCP parameter set, and besides, it depends on the cohort of the patient and treatment technique used. It is essential to use a more accurate predictor while comparing treatment plans and taking a clinical decision based on dosimetric benefit and absolute NTCP reduction (∆NTCP) [17].

Hence, the research questions of the present in-silico planning comparison study were: 1) In comparison with IMRT, can the dose delivered to GI-OAR for LAUPC of the head be lowered using ro-IMPT? 2) what is the anticipated clinical advantage of this GI-OARs sparing? To answer these questions, we performed an NTCP radiobiological model-based comparison study between IMRT and ro-IMPT for LAUPC of the head with EDR, and we hypothesized that ro-IMPT could reduce GI-OAR toxicity.

## Methods

The clinicopathological data of patients were reviewed from the hospital’s medical records. With the approval from the Institute Research Ethics Committee (Reference number: 2017–440), for this study, we identified nine locally-advanced pancreatic ductal adenocarcinoma (LAUPC) of the head patients with T4 disease (encasing superior mesenteric artery or celiac axis) as per the 7th edition of the American Joint Committee on Cancer (AJCC) staging manual from 2015 to 2018. The treatment planning computed tomography (CT) in the supine position was obtained for these 9 patients with a 3 mm slice thickness. Each patient was re-planned for IMRT and ro-IMPT.

### Target volume and OAR delineation

Target volume and OARs were contoured on IV contrast CT simulation scans. Gross disease and clinically apparent nodes were included as gross tumor volume (GTV); however, the elective nodal region was not included in the target volume [18, 19]. For this study, the GTV to clinical target volume (CTV) was given margin of 0.5 cm, and the CTV was edited at the interface of the GI-OARs, and as per the previously published phase I/II dose-escalation studies, CTV to planning treatment volume (PTV) was given isotropic expansion margin of 0.5 cm as shown in Additional Figure 1 [6]. In this study for reducing the motion to estimate maximum potential benefit, it was supposed that all patients would be treated using breath-hold technique [16, 20].

The OARs were contoured for all patients, and it includes the whole stomach, the duodenum was from pylorus till ligament of Treitz, bilateral kidney, small bowel loops, liver, and spinal cord. The small bowel loops were contoured 2 cm superior-inferiorly to PTV [15]. The whole liver was contoured, including the vessels and intraductal biliary system. The organ contour “Stoduo” was created, which combines stomach and duodenum for comparison with previously published studies [21].

### Dose prescription and OARs constraints

The prescription dose was 59.4GyE at 1.8GyE/ fraction in 33 fractions [10, 11]. The proton beam output was modulated with relative biological effectiveness (RBE) of 1.1 [22]. As all tissues are presumed to have nearly the same RBE, the dose stated in GyE is directly in comparison with the photon doses. The planning goal for IMRT and ro-IMPT was at least 100% of GTV receives ≥95% of the dose, at least ≥98% of CTV receives ≥95% of the dose, and 0% volume of CTV receives < 107% of the prescribed dose. Besides, our goal during IMRT was also to provide adequate PTV coverage of at least 95% of PTV receiving 95% of the dose. One physicist designed all IMRT plans, and all ro-IMPT plans were created by another physicist and were checked by two physicians.

### The OARs constraints were

For stomach wall, ≤16 cc receive 50GyE, ≤ 10%volume receive 50GyE, ≤15% volume receive 45GyE, and 0.1 cc receive ≤60GyE [21, 23]. For duodenum, ≤45% volume receive 25GyE, 1 cc receive ≤55GyE, and 0.1 cc receive ≤60GyE [9, 24]. For small bowel loops, ≤ 10%volume receive 50GyE, ≤15% volume receive 45GyE, ≤5% volume receive 54GyE, and 0.1 cc volume receive ≤60GyE [23]. For Kidneys, mean dose ≤18GyE and V23GyE < 30%. The mean liver goal was ≤30GyE, V30GyE ≤ 50%, V35GyE ≤ 33%, and 0.1 cc of spinal cord receive <45GyE.

### IMRT and ro-IMPT planning, beam configuration, and optimization

For each patient, two plans were generated (IMRT and ro-IMPT). The non-coplanar 6 beam IMRT plan was made using Raystation v6.2 (Raysearch Laboratories, Stockholm, Sweden) treatment planning system (TPS).

**Fig. 1:**
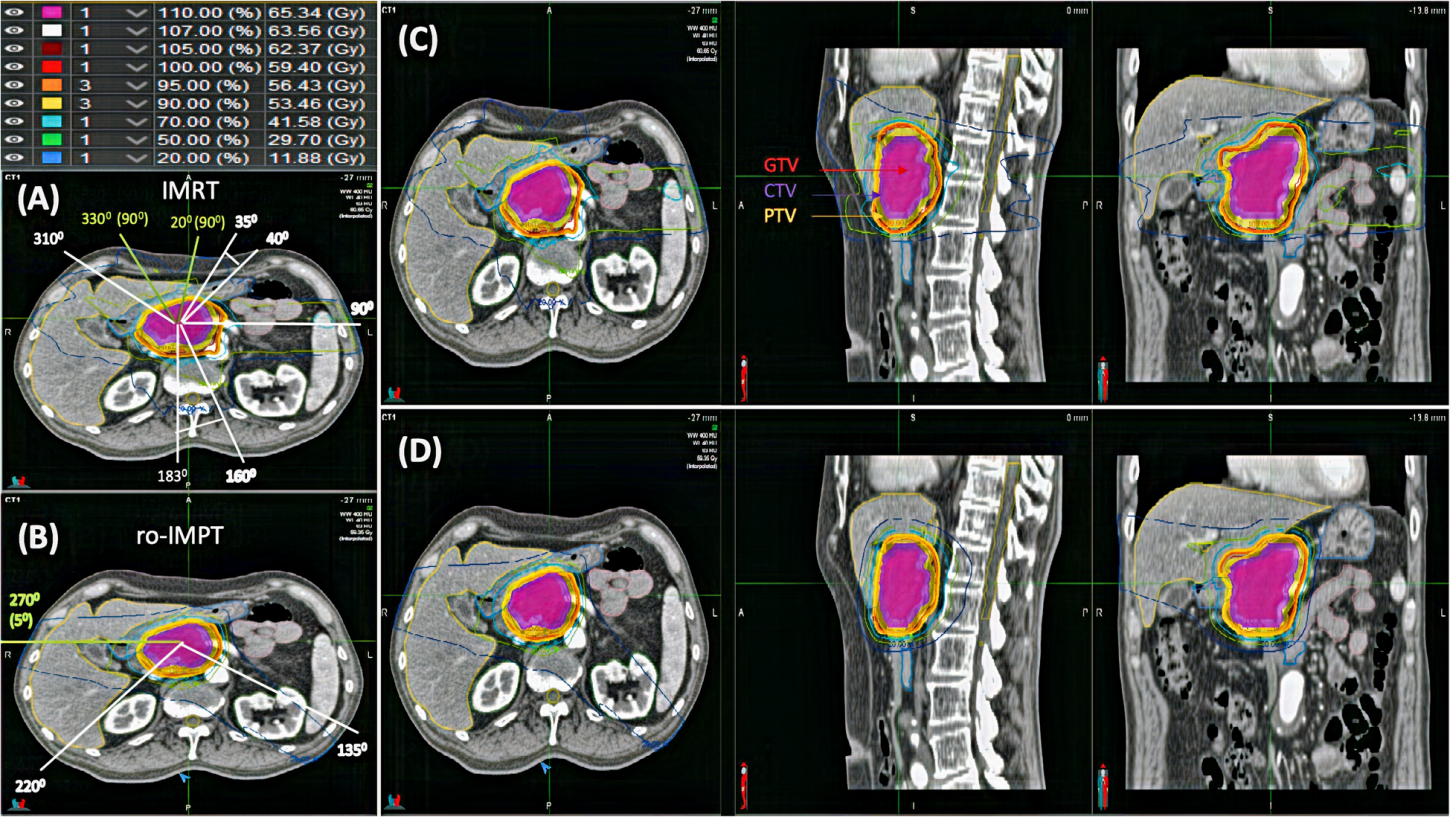
Overview of the beam configuration (**a** and **b**) and axial, sagittal, and coronal CT slices showing dose distribution of IMRT (**c**) and ro-IMPT (**d**) treatment plan. The range of beam direction and couch angle in all patients is given per beam direction. Non-coplanar beam direction is marked in green, and co-planar beam direction in white

Non-coplanar CTV-based robust multifield optimization IMPT plan was made using Eclipse (v15.1) TPS (Varian Medical System, Inc., Palo Alto, CA). All the ro-IMPT plan was delivered using 3 beams, 2 co-planar beams (135^0^ and 220^0^), and one non-coplanar beam (gantry at 270^0^ with couch at 5^0^) as shown in Fig. 1. The two posterior oblique fields were used so that the proton beam minimally intersects with high uncertainty tissue, in particular, the diaphragm and bowel, and is deemed to be more robust against intra-fractional motions. Also, to reduce the dosimetric impact of organ filling and motion uncertainty, a right lateral field was added through the liver [10, 14, 25]. A more detailed description of IMRT and ro_IMPT planning, beam configuration, and optimization is presented in Additional file 1.

### Plan evaluation

For IMRT and ro-IMPT plan evaluation, the DVH of targets (GTV and CTV) and OARs were generated on nominal dose distributions. The IMRT and ro-IMPT plans were compared for target homogeneity and conformity. The target volume and OARs DVH physical dose parameters were documented. Homogeneity is defined by the dose distribution consistency of a plan throughout the target volume. The RTOG formula [(D2%-D98%/D50%)] was used to computed HI (Homogeneity index). Where D2%, D98%, and D50% are the dose received by 2, 98, and 50% of the target volume. The conformation number (CN) formula for CTV [(CTV95) [2]/ (CTV*V95) was used to define conformity around the CTV. Where CTV is a target volume, CTV95 is target volume covered by 95% of reference isodose, and V95 is a volume of 95% isodose. As CN value approaches 1, the plan is deemed to be more conformal, and plan with CN 0 indicates the total absence of conformity or a huge volume of irradiation compared to the target volume.

### Dose-volume data and Normal tissue complication probability

Coverage of target volume and various dose-volume parameters were assessed. The Digital Imaging and Communications in Medicine (DICOM) standard RT doses from IMRT and ro-IMPT plan were transferred to MIM (v6.86, MIM Software Inc., Cleveland, United States). Before NTCP calculation, the linear-quadratic (LQ) equation with α/β=4 (for the stomach, duodenum, small bowel, and stoduo) was used to convert the cumulative physical dose into an equivalent dose of 2Gy (EQD2) per fraction.

The radiobiological Lyman-Kutcher-Butcher (LKB) model was used to computed NTCP for GI toxicity endpoints using parameters from Pan et al., Burman et al., and Holyoake et al., as shown in Table 1 [26–29]. Computed NTCP values were used in a relative sense for comparison between ro-IMPT and IMRT. The RADBIOMOD Visual Basic for Application (VBA) software was used to calculate NTCP values from EQD2 DVH’s ASCII files [30]. The absolute NTCP reduction (∆NTCP_IMRT – ro-IMPT_) and quantitative relative risk (RR = NTCP_ro-IMPT_/NTCP_IMRT)_ ratios for GI-OARs was also computed.

**Table 1:** Normal tissue complication probability (NTCP) LKB model parameters used in biological evaluation of IMRT and ro-IMPT plans

Gastrointestinal OAR (Reference)	TD_50_ (Gy) (range)	m (range)	n (range)	Endpoint
Stomach wall (Pan et al.) [26]	62	0.30	0.07	Gastric bleed
Stomach wall (Burman et al.) [27]	65	0.14	0.15	Ulceration/Perforation
Duodenum (Pan et al.) [26]	180	0.49	0.12	Gastric bleed
Duodenum (Holyoake et al.) [28]	299.1	0.51	0.193	Grade ≥ 3 GI toxicity
Small bowel loops (Burman et al.) [27]	55	0.16	0.15	Obstruction/Perforation
StoDuo (Pan et al.) [26]	52.5	0.35	0.21	Gastric bleed

*Abbreviations*: *OAR* organ at risk, *TD*_*50*_*(Gy)* dose at which there is 50% chance of complication, *m* slope of dose-response curve, *n* dose-volume relationship

### Statistical analysis

The mean and standard deviation (SD) was used to describe all continuous variables. The non-parametric Wilcoxon sign rank exact test provides an estimate of statistical significance between techniques. Two-sided *P*-value < 0.05 was considered to be statistically significant. R statistical software version 3.6.3 (R commander EZR version 2-6.2) was used for all statistical analysis.

## Result

### Target dose parameters evaluation

**Fig. 2:**
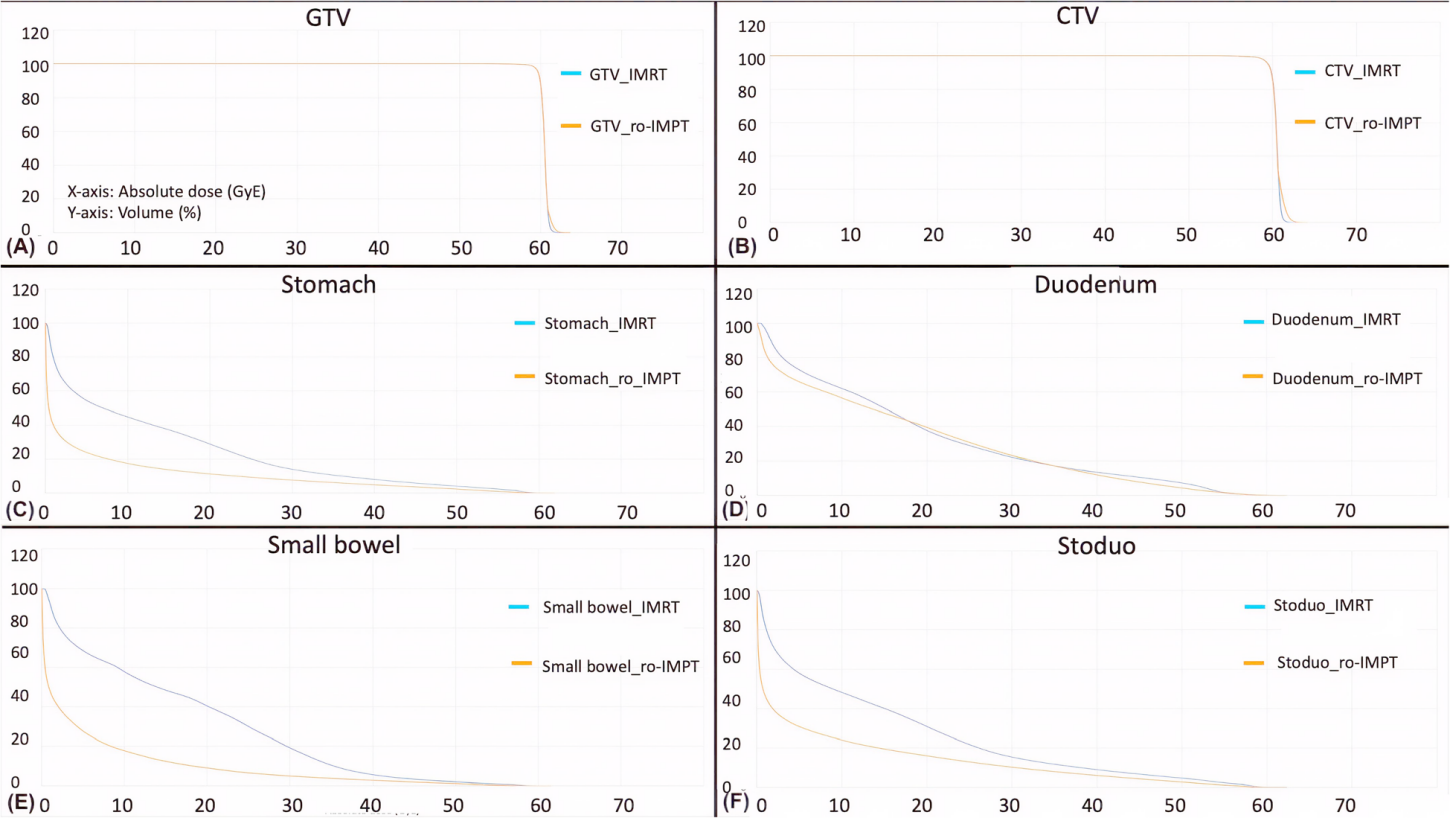
Comparisons of average cumulative DVH curve for target volume (GTV and CTV), stomach, duodenum, small bowel, and stoduo using IMRT and ro-IMPT plans. Average DVH are shown for each cohort of plans (IMRT and ro-IMPT). Radiation dose is shown along the X-axis and cumulative volume receiving at least dose is plotted on Y-axis

The patient characteristics are shown in Table 2. The median GTV, CTV, and PTV volumes were 37.6 cc (range, 22.9 to 54.3 cc), 76.1 cc (range, 49.3 to 99.8 cc), and 135.5 cc (range, 93.9 to 178.0 cc) respectively. IMRT and ro-IMPT dose distribution for one representative patient is shown in Fig. 1. Target coverage for all plans met the required goal for the GTV (D_100%_ ≥ 95%) and CTV (V_95%_ ≥ 98%), and the result for target coverage is shown in Fig. 2 (a and b). CTV CN for ro-IMPT plan show significantly (*P* = 0.004) better conformation of the dose; as a result, a lower percentage of the body outside the CTV was irradiated to high doses with ro-IMPT than with IMRT.

**Table 2:** Patient characteristics

Cases	Age (years)	Sex	TNM staging^†^	GTV volume (cc)	CTV volume (cc)	PTV volume (cc)
1	56	Male	T4N1	38.9	82.7	148.5
2	81	Male	T4N0	37.6	76.1	135.5
3	55	Male	T4N0	51.5	99.8	178.0
4	64	Male	T4N0	22.9	49.3	93.9
5	77	Male	T4N0	42.6	88.6	154.1
6	70	Male	T4N0	31.1	65.5	126.6
7	78	Female	T4N0	26.8	51.2	97.8
8	59	Female	T4N0	32.7	63.0	112.9
9	72	Female	T4N1	54.3	98.9	168.3

*Abbreviation*: *GTV* Gross tumor volume, *CTV* Clinical target volume, *PTV* Planning target volume

^†^ Staging was done using American Joint Committee of Cancer guideline (7th edition manual, 2010)

### Dose delivered to OARs

Physical dosimetric OAR DVH parameters were significantly lower in the ro-IMPT plan in comparison to IMRT, in low to high dose range (V10GyE to V55GyE) for the stomach, small bowel, and stoduo (Table 3 and Fig. 2 c, e, and f). For stoduo, V50GyE was 7.91 ± 4.4 cc with ro-IMPT vs. 12.9 ± 5.6 cc with IMRT (*P* = 0.007; Table 3). For duodenum, ro-IMPT delivered a significantly lesser dose in low dose area (≤10Gy) in comparison to IMRT (Table 3 and Fig. 2d).

**Table 3:** Target volumes and OARs physical dosimetric parameters and comparative analysis between IMRT and ro-IMPT plans

Dosimetric Parameters	IMRT ((Mean ± SD)	ro-IMPT (Mean ± SD)	*P*-value IMRT vs. ro-IMPT
CTV coverage			
CTV HI	0.04 ± 0.01	0.07 ± 0.01	0.004*
CTV CN	0.43 ± 0.03	0.58 ± 0.05	0.004*
Stomach			
V55GyE	2.3 ± 1.0%	1.0 ± 0.9%	0.01*
V50GyE in cc	9.8 ± 4.9 cc	5.5 ± 4.6 cc	0.01*
V50GyE	4.0 ± 2.1%	2.3 ± 2.0%	0.01*
V45GyE	5.8 ± 3.2%	3.5 ± 3.1%	0.01*
V40GyE	7.8 ± 4.7%	5.9 ± 3.7%	0.01*
V30GyE	13.8 ± 9.2%	7.5 ± 5.6%	0.007*
V20GyE	28.7 ± 15.9%	11.1 ± 7.6%	0.004*
V10GyE	44.7 ± 18.0%	17.3 ± 12.0%	0.004*
D_0.1cc_	56.4 ± 8.9 GyE	56.1 ± 8.3 GyE	0.44
Duodenum			
V55GyE	1.8 ± 2.0%	1.2 ± 0.7%	0.36
V50GyE	7.5 ± 6.7%	3.6 ± 2.4%	0.11
V40GyE	13.7 ± 11.8%	11.4 ± 6.5%	0.16
V30GyE	22.7 ± 12.4%	22.9 ± 11.0%	0.73
V25GyE	29.5 ± 13.6%	30.5 ± 13.3%	0.09
V20GyE	39.4 ± 15.5%	39.4 ± 16.8%	0.49
V10GyE	62.8 ± 21.3%	56.5 ± 22.3%	0.003*
D_0.1cc_	56.5 ± 5.6 GyE	55.3 ± 6.4 GyE	0.17
D_1cc_	49.9 ± 7.1 GyE	49.5 ± 8.1 GyE	0.42
Small Bowel			
V54GyE	1.2 ± 1.4%	0.5 ± 0.8%	0.02*
V50GyE	2.2 ± 2.5%	1.2 ± 2.0%	0.02*
V45GyE	3.5 ± 4.2%	2.1 ± 3.5%	0.02*
V40GyE	5.7 ± 6.2%	2.9 ± 4.7%	0.01*
V30GyE	19.3 ± 17.0%	5.1 ± 6.9%	0.004*
V20GyE	40.5 ± 19.6%	9.1 ± 9.3%	0.003*
V10GyE	57.9 ± 15.1%	17.9 ± 12.5%	0.004*
D_0.1cc_	54.8 ± 7.9GyE	51.0 ± 10.9GyE	0.057
StoDuo			
V55GyE	2.3 ± 1.2%	0.9 ± 0.8%	0.004*
V50GyE in cc	12.9 ± 5.6 cc	7.91 ± 4.4 cc	0.007*
V50GyE	4.5 ± 2.4%	2.5 ± 1.9%	0.01*
V40GyE	8.7 ± 4.6%	5.9 ± 3.7%	0.02*
V30GyE	15.2 ± 8.3%	10.1 ± 5.6%	0.03*
V20GyE	30.6 ± 13.5%	15.9 ± 7.8%	0.01*
V10GyE	48.0 ± 15.0%	23.9 ± 12.7%	0.004*
Kidneys			
Mean dose (GyE)	6.01 ± 1.16 GyE	9.82 ± 2.80 GyE	0.004*
V23GyE	0.04 ± 0.07%	6.6 ± 6.7%	0.02*
Liver			
Mean dose (GyE)	6.53 ± 3.2 GyE	5.64 ± 2.5 GyE	0.09
V35GyE	3.0 ± 2.2%	1.8 ± 1.2%	0.02*
V30GyE	4.2 ± 3.0%	2.6 ± 1.6%	0.03*
Spinal Cord			
D_0.1cc_	20.8 ± 1.9 GyE	7.27 ± 4.3 GyE	0.004*

*Abbreviation*: *IMRT* Intensity-modulated radiotherapy, *ro-IMPT* Robust Intensity-modulated proton therapy, *CTV* Clinical target volume, *CN* Conformation number, *HI* Homogeneity index, *GyE* Gray equivalent, *cc* cubic centimeter, *V*_*(X)%*_ percentage volume of OAR at or above “X” GyE, *D*_*(X)cc*_ GyE dose of OAR to “X” cc volume, *SD* Standard deviation

*Significant (*P* < 0.05)

For liver, V35GyE and V30GyE were significantly lower in ro-IMPT in comparison to IMRT. In contrast, the kidneys D_mean_ and V23GyE were significantly higher with ro-IMPT (Table 3).

For the spinal cord, D_0.1cc_ was 7.27 ± 4.3GyE with ro-IMPT vs. 20.8 ± 1.9GyE with IMRT (*P* = 0.004; Table 3).

The change in dose to OARs with robustness on the CTV at its worst iteration compared to nominal doses is shown in Additional figures 3, 4, and 5. The dose constraint was met for all OARs with robustness on the CTV in worst-case iteration except for the duodenal constraints, V25GyE ≤ 45% for three patients, D_0.1cc_ ≤ 60Gy for one patient, and D_1cc_ ≤ 55Gy for six patients as shown in Additional figure 3.

### NTCP analysis

**Fig. 3:**
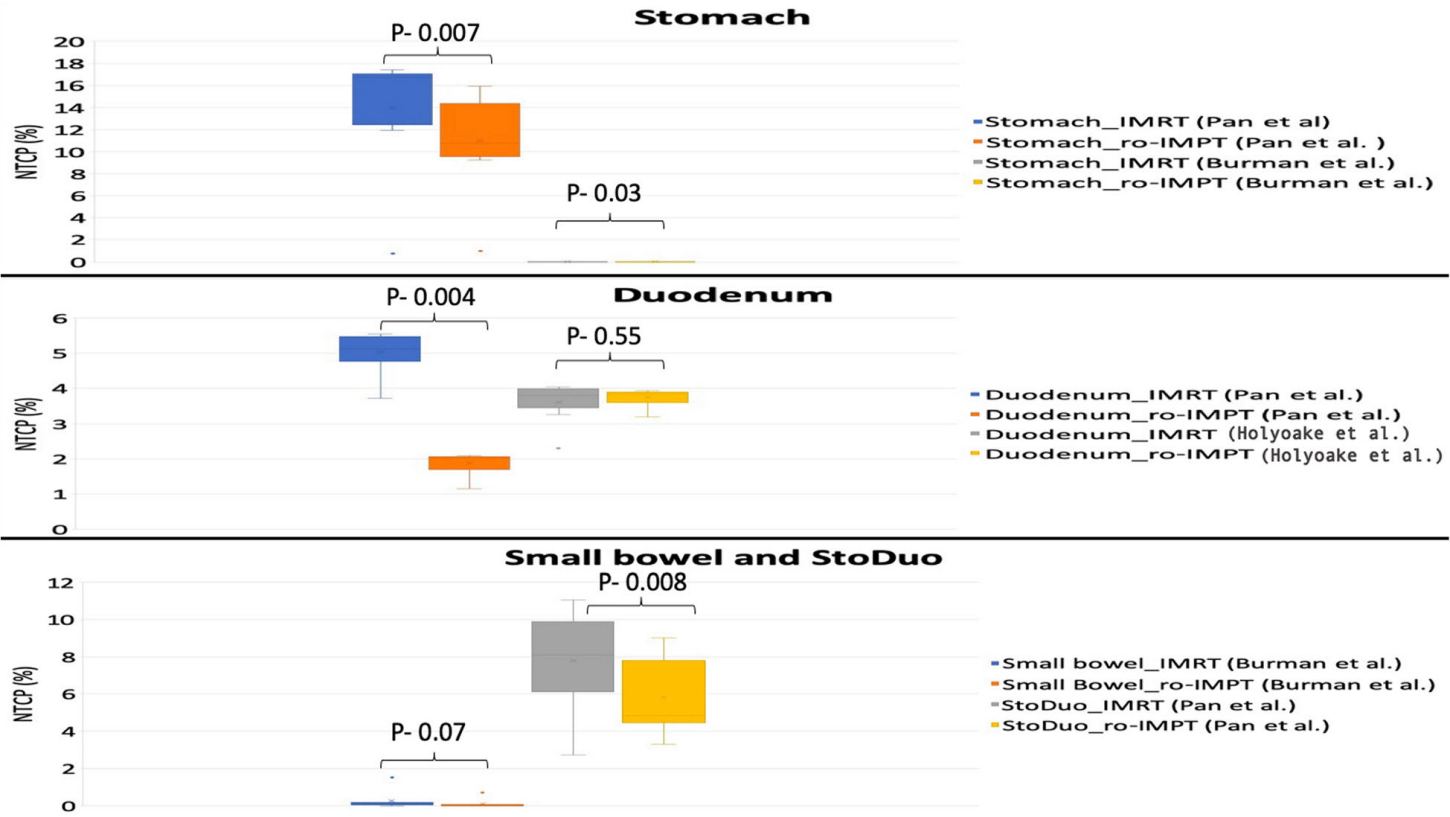
Box and whisker plot of NTCP (%) comparison for gastrointestinal OARs (Stomach, duodenum, small bowel, and stoduo) using Pan et al., Burman et al., Holyoake et al., LKB model parameters between IMRT and ro-IMPT treatment plans

As reported in Table 4 and Fig. 3, the probability of gastric ulceration/perforation and gastric bleed was significantly worse in the IMRT plans in comparison to ro-IMPT plans according to the model of Pan et al. (stomach, duodenum, and stoduo) and Burman et al. (stomach) [26, 28]. The NTCP value for small bowel was not significantly different in two irradiation techniques (Table 4).

**Table 4:** Normal tissue complication probability (NTCP), Relative risk (RR) ratio, number of patients with ∆NTCP_IMRT – ro-IMPT_ in specific range for gastro-intestinal OARs toxicity

Gastro-intestional OAR	NTCP (%)		*P*-value IMRT vs. ro-IMPT	Relative risk (RR) ratio (Mean ± S.D)	∆NTCP_IMRT – ro-IMPT_ (n/N)		
	IMRT (Mean ± S.D)	ro-IMPT (Mean ± S.D)			≤5%	> 5 to ≤10%	> 10%
Stomach wall							
U/P (Burman et al.)	0.02 ± 0.01%	0.01 ± 0.01%	0.03*	0.16 ± 0.24	9/9	0/9	0/9
GB (Pan et al.*)*	13.97 ± 5.33%	10.55 ± 4.10%	0.007*	0.81 ± 0.19	6/9	3/9	0/9
Duodenum							
GB (Pan et al.)	5.02 ± 0.57%	1.87 ± 0.31%	0.004*	0.37 ± 0.28	9/9	0/9	0/9
Grade ≥ 3 GI toxicity (Holyoake et al.)	3.60 ± 0.54%	3.74 ± 0.24%	0.55	1.1 ± 0.22	9/9	0/9	0/9
Small bowel loops							
O/P (Burman et al.)	0.26 ± 0.47%	0.10 ± 0.23%	0.07	0.24 ± 0.22	9/9	0/9	0/9
StoDuo							
GB (Pan et al.)	7.81 ± 2.53%	5.67 ± 2.15%	0.008*	0.76 ± 0.22	7/9	2/9	0/9

*Abbreviations*: *IMRT* Intensity-modulated radiotherapy, *ro-IMPT* Robust Intensity-modulated proton therapy, *GB* Gastric bleed, *U/P* ulceration/perforation, *O/P* obstruction/perforation, *OAR* organ at risk; *Relative risk (RR) ratio* NTCP_ro-__IMPT_/NTCP_IMRT_; ∆NTCP_IMRT – ro-IMPT_, *n/N* number of patients with specific ∆NTCP range/total number of patient (where *N* = 9), *SD* Standard deviation; NTCP derived using parameter from Pan et al., Burman et al., and Holyoake et al.

*Significant (*P* < 0.05)

**Fig. 4:**
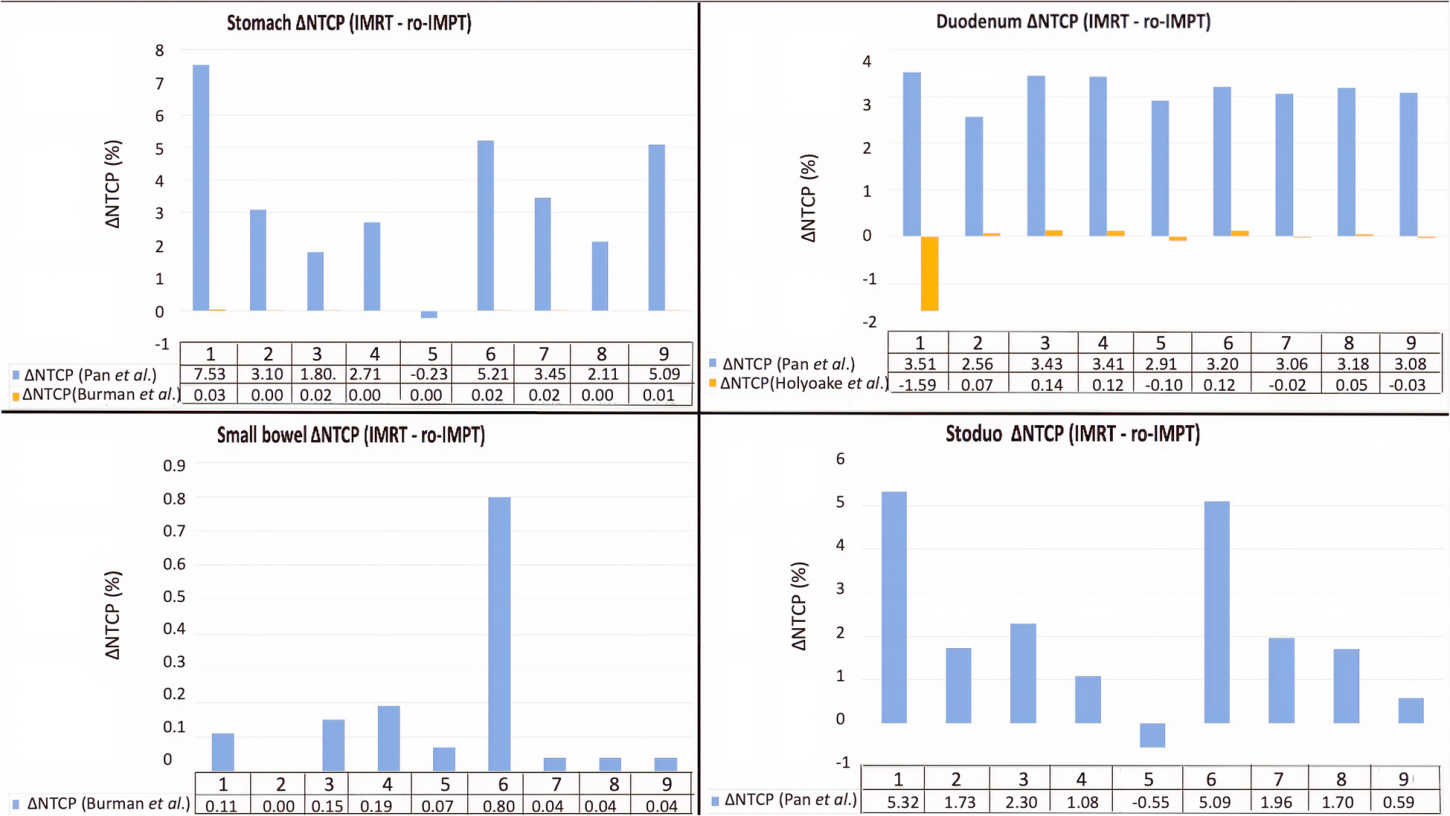
Bar graph of NTCP reduction (∆NTCP_IMRT – ro-IMPT_) for GI-OARs (Stomach, duodenum, small bowel, and stoduo) of each patient using Pan et al., Burman et al., Holyoake et al., LKB model parameters between IMRT and ro-IMPT treatment plans

The ∆NTCP_IMRT – ro-IMPT_ of ≥5 to < 10% was seen for endpoint gastric bleeding of the stomach (3 patients) and stoduo (2 patients) as shown in Table 4 and Fig. 4. The NTCP mode application to GI OARs has demonstrated an overall GI toxicity relative risk reduction (RR < 1) except for endpoint grade ≥ 3 toxicity for duodenum (RR > 1) for all ro-IMPT plans in comparison to IMRT plans (Table 4). According to the considered toxicity endpoint for the stomach, small bowel, and stoduo, the RR values ranged from 0.16 to 0.81 (Table 4).

## Discussion

Our study demonstrates a significant GI-OARs sparing benefit using ro-IMPT over IMRT plans in EDR for LAUPC of the head with better target conformation. Clinically acceptable plan with target coverage goal and OAR dose constraint were made for all patient with both the techniques. To our knowledge, this is the first radiobiological model-based comparative study in LAUPC of the head with EDR to assess the potential radiobiological-based clinical implication of ro-IMPT in reducing GI-OARs toxicity.

Dose to stomach (V50GyE ≤ 16 cc), stoduo (V50GyE ≤ 33 cc), and duodenum (V25GyE ≤ 45%, D_1cc_, and D_0.1cc_) were below the threshold predicting the low risk of grade ≥ 2 acute GI toxicity and upper GI bleeding in both the radiation delivery technique [9, 21, 24]. Also, in contrast to the study by Thompson et al. and Bouchard et al., the current study demonstrates that the ro-IMPT plan significantly decreases volume receiving intermediate and higher dose for stomach and small bowel, in addition to a decrease in doses < 30 Gy [15, 16]. Using multi-field optimization ro-IMPT in our study compared with single field optimization in Thompson et al., and reducing the beam penumbra by using a small PBS spot size, explains this dissimilarity in intermediate and high doses region. This result corresponds with the study by Jethwa et al., using ro-IMPT in pancreatic cancer, which might facilitate EDR for cases in which OAR are closely surrounding the GTV in almost all the directions [31].

The study published by Thompson et al., reported that the proton therapy, in comparison to IMRT, substantially reduces the dose in low-intermediate dose range [16]. However, the clinical implication of their result is uncertain. In contrast to their study, we performed an absolute NTCP reduction and relative risk (RR) assessment for GI-OARs. In our study, the ∆NTCP_IMRT – ro-IMPT_ of ≥5 to < 10% was seen for the gastric bleeding endpoint of Pan et al., for GI-OARs stomach in 3 patients (33%) and stoduo in 2 patients (22%). The ro-IMPT plans reduce the relative risk of toxicity for the stomach (gastric bleeding, ulceration, and perforation), small bowel (obstruction and perforation), duodenum (gastric bleeding), and stoduo (gastric bleeding) for all patients.

For small bowel, the significant lesser dose volume in high, intermediate, and low dose range for ro-IMPT did not translate into a decrease in NTCP. This shows that, even though a reduction of dose to OAR been excellent, a statistically significant dosimetric difference may not interpret into clinically considerable differences. The use of a radiobiological NTCP model and NTCP-based quantitative relative risk assessment simplifies the task for different planning technique comparisons. It is more robust than DVH parameters for investigation of GI-OARs related toxicity in spite of the uncertainties in NTCP model parameters.

Our present study has several limitations, and the potential limitation is the use of photon-derived tissue NTCP models. To authenticate the results of this study, a large and reliable clinical outcome data are needed. Because such data are lacking, possibly a significant change in model-based toxicity and uncertainties are seen when these radiobiological parameters are used to define the advantage between radiation technique. This can impact the absolute NTCP values, and thus the ∆NTCP. Although clinical validation of these NTCP models was out of the scope of this study, the relative NTCP comparison must be meaningful. The NTCP model selected in our study was generated based on similar patient cohorts and treatment for upper gastrointestinal tumors. Cautious interpretation of these results is essential because it may be affected by model uncertainties.

In LAUPC of the head, while designing a proton beam with the posterior field, the GI-OARs may be positioned inside the distal Bragg peak. Caution should be taken when delivering a dose for LAUPC of the head given its proximity to the duodenum, and the end range uncertainty that must be taken into account is particularly crucial for dose escalation strategy. An increase in effective biological dose in these organs may result in a higher risk of adverse events. In most cases, this increase in dose can be accounted for with small alteration to the physical dose or treating at a lower total physical dose. In certain cases, it is advantageous to add a field to decrease the overall biological effective dose [32]. Our study had not taken into consideration the effectiveness of variable RBE for protons assuming interpatient variability of α/β [33]. A significant uncertainty with the NTCP values and ∆NTCP can occur, as a result of considerable uncertainty with the RBE variation. This uncertainty may cause a substantial increase in dose to the OARs if the OARs are close to the target volume [34].

The target overlap with GI-OARs could restrict EDR for pancreatic cancers. The ITV is generated to account for pancreatic tumor motion with respiration, which could hinder safe EDR, and respiratory gating could benefit such cases. In advanced pancreatic cancer, regional recurrence remains uncommon in comparison to the local and distant recurrence [19]. Hence, in our study, we opted for EDR to a limited CTV without elective nodal irradiation (ENI) to reduce the risk of an adverse event. Inclusion of ENI in our study would have resulted in an increase in the irradiated volume of the stomach, duodenum, small bowel, kidney, liver, and spinal cord using IMRT in comparison to ro-IMPT as demonstrated by Jethwa KR et al.*,* comparing ro-IMPT with VMAT [31].

A comparison of ro-IMPT plans was carried out for nominal dose distributions supposing an idealized patient setup model based on a single CT scan, wherein the anatomical and geometric changes were not taken into consideration. The GI-OARs are an expansible and movable organ, and as a result, determining the accurate dose-volume constraints is quite challenging [35]. Therefore, well-defined image guidance protocol and adaptive treatment strategy are essential during the clinical implementation of ro-IMPT. Despite that, with this approach, further uncertainties on dose distributions are being introduced through deformable image registration [36]. The ro-IMPT plan optimized and evaluated considering the setup and range uncertainties is generally robust for non-rigid anatomical changes visualized on a repeat CT scans [37]. Nevertheless, according to our understanding, the biases of organ motion, positioning, and respiration tend to occur among patients who are treated using both RT techniques, and hence, should not undermine the comparison of the GI-OARs DVHs.

In future studies, for a better comparison of proton and photon plans, proton plans should be calculated, taking variable RBE into accounts [38]. Investigating the use of image registration and fusion algorithm for dose mapping may be necessary to precisely compute the dose delivered to GI-OARs to confirm the eminence of ro-IMPT plan during radiation.

In conclusion, given the smaller sample size and design of our study, ro-IMPT can potentially provide a substantial decrease in GI-toxicity risk for LAUPC of the head in EDR in comparison to IMRT. The quantitative risk evaluation also supports the potential clinical benefit of EDR IMPT for LAUPC of head due to the lower risk of GI morbidity. The result of our study using EDR ro-IMPT should be considered as hypothesis-generating for future clinical trials and research to verify the expected risk reduction in GI toxicity.

## Supplementary information

**Additional file 1: Figure 1.** Axial, sagittal, and coronal CT image of one representative patient with gross tumor volume (GTV), clinical target volume (CTV), planning treatment volume (PTV), and organs at risk (OARs) contours.

**Additional file 2: Figure 2.** Plan uncertainty DVH for one representative patient to quantify robustness of the ro-IMPT treatment plan (Dose coverage in each worst-case scenario met the CTV criteria of V_95%_ ≥ 98% and D_0%_ < 107%). Radiation dose in Gy and % is shown along the X-axis and structure volume (%) along the Y-axis.

**Additional file 3: Figure 3.** Duodenal dose with robustness analysis on the CTV, at its worst iteration. In this figure the nominal and worst-case scenario value for the duodenum are shown. Dashed lines represent the dose constraints. (Abbreviations: D_Xcc_: dose received by Xcc volume; V_xGy_: volume receiving x-Gy).

**Additional file 4: Figure 4.** Stomach and small bowel dose with robustness analysis on the CTV. In this figure the nominal and worst-case scenario value for stomach and small bowel are shown. Dashed lines represent the dose constraints. (Abbreviations: D_Xcc_: dose received by Xcc volume; V_xGy_: volume receiving X-Gy).

**Additional file 5: Figure 5.** Kidneys, liver, and spinal cord dose with robustness analysis on the CTV at its worst iteration. In this figure the nominal and worst-case scenario value for Kidneys, liver, and spinal cord are shown. Dashed lines represent the dose constraints. (Abbreviations: D_mean_: mean dose; D_Xcc_: dose received by X cc volume; V_xGy_: volume receiving X-Gy).

**Additional file 6.**

## Data Availability

The datasets supporting the conclusion of this article are included within the article.

## Abbreviations

LAUPC: Locally advanced unresectable pancreatic cancer;
CRT: Concurrent chemoradiotherapy
IMRT: Intensity-modulated radiotherapy
GI: Gastrointestinal
DS: Double scatter proton beam therapy
PBS: Pencil beam scanning proton therapy
PBT: Proton beam therapy
CTV: Clinical target volume
PTV: Planning treatment volume
OARs: Organ at risk
GyE: Gray equivalent
IMPT: Intensity-modulated proton radiotherapy
ro-IMPT: Robust intensity-modulated proton radiotherapy
RBE: Relative biological equivalent
NTCP: Normal tissue complication probability
∆NTCP: NTCP reduction
DX%: Dose received by X% of the volume
VXGy: Volume receiving X-Gy
DVH: Dose-volume histogram
CN: Conformation number
HI: Homogeneity index
LKB: Lyman-Kutcher-Burman
*p*-value: Probability value
RR: Relative risk
