# Supplementary figures and images for: Radiobiological model-based approach to determine the potential of dose-escalated robust intensity-modulated proton radiotherapy in reducing gastrointestinal toxicity in the treatment of locally advanced unresectable pancreatic cancer of the head
Source: Radiat Oncol. 2020 Jun 22;15:157. doi: 10.1186/s13014-020-01592-6 (PMC7310413; doi:10.1186/s13014-020-01592-6)

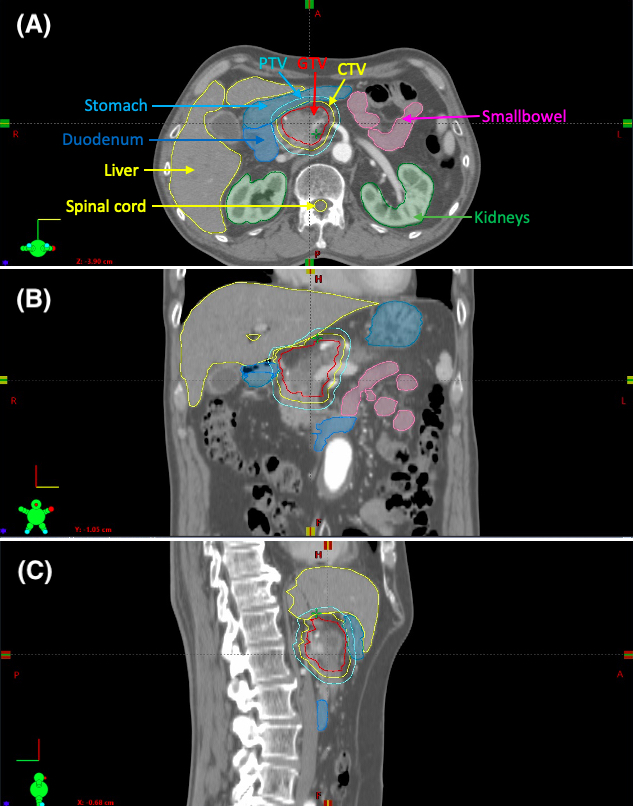

Supplement: Supplementary file 1 — Additional file 1: Figure 1. Axial, sagittal, and coronal CT image of one representative patient with gross tumor volume (GTV), clinical target volume (CTV), planning treatment volume (PTV), and organs at risk (OARs) contours. [file 13014_2020_1592_MOESM1_ESM.jpg]

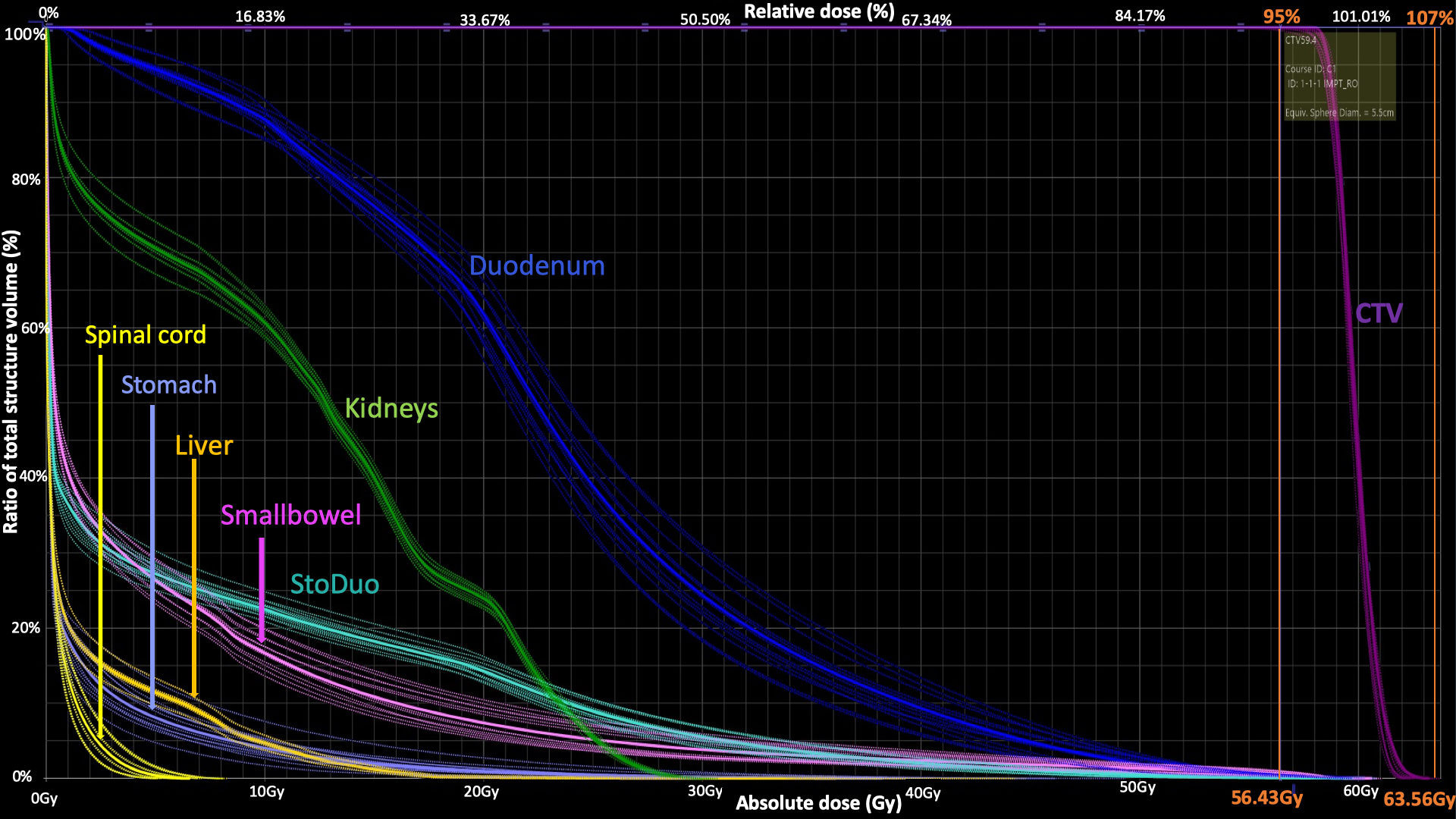

Supplement: Supplementary file 2 — Additional file 2: Figure 2. Plan uncertainty DVH for one representative patient to quantify robustness of the ro-IMPT treatment plan (Dose coverage in each worst-case scenario met the CTV criteria of V95% ≥ 98% and D0% < 107%). Radiation dose in Gy and % is shown along the X-axis and structure volume (%) along the Y-axis. [file 13014_2020_1592_MOESM2_ESM.jpg]

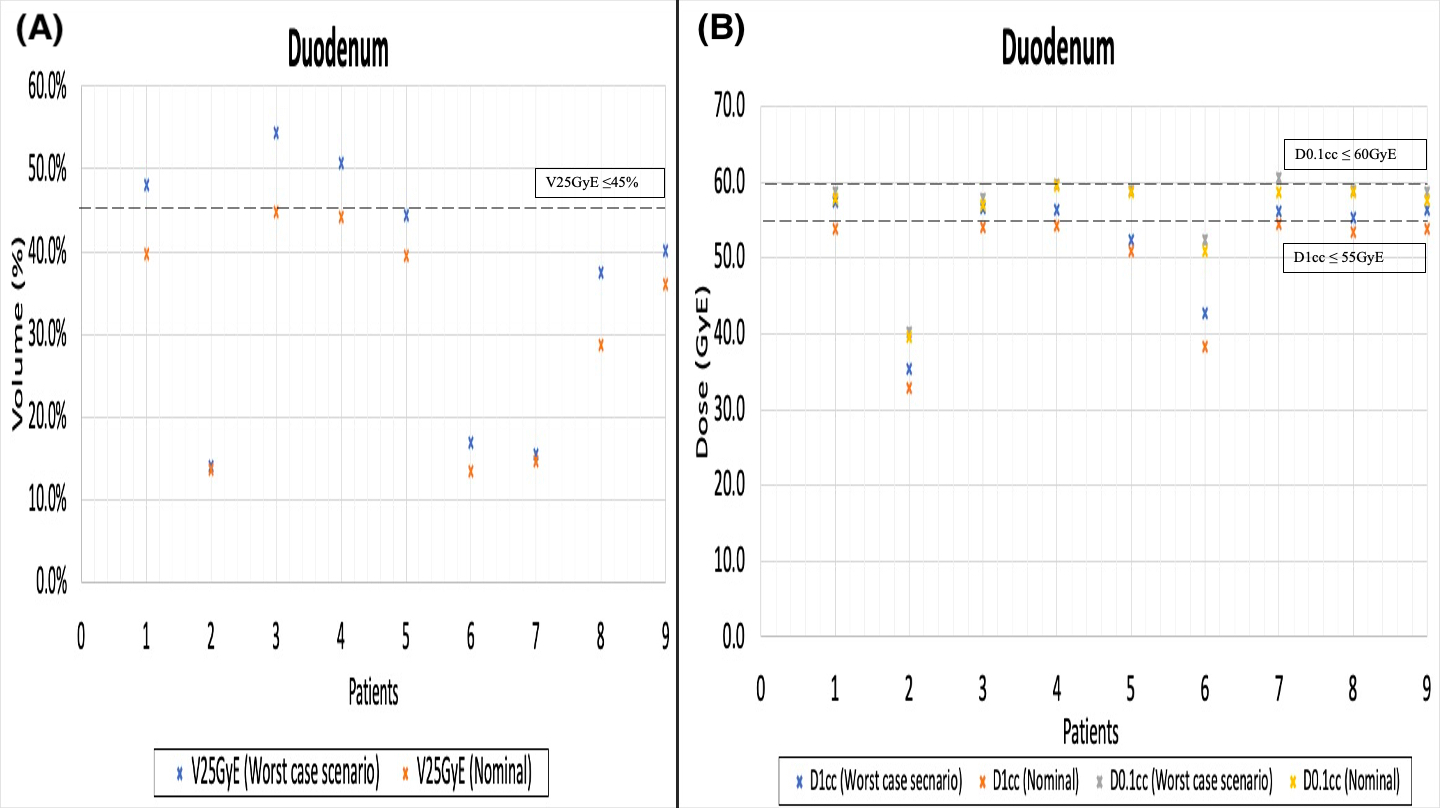

Supplement: Supplementary file 3 — Additional file 3: Figure 3. Duodenal dose with robustness analysis on the CTV, at its worst iteration. In this figure the nominal and worst-case scenario value for the duodenum are shown. Dashed lines represent the dose constraints. (Abbreviations: DXcc: dose received by Xcc volume; VxGy: volume receiving x-Gy). [file 13014_2020_1592_MOESM3_ESM.jpg]

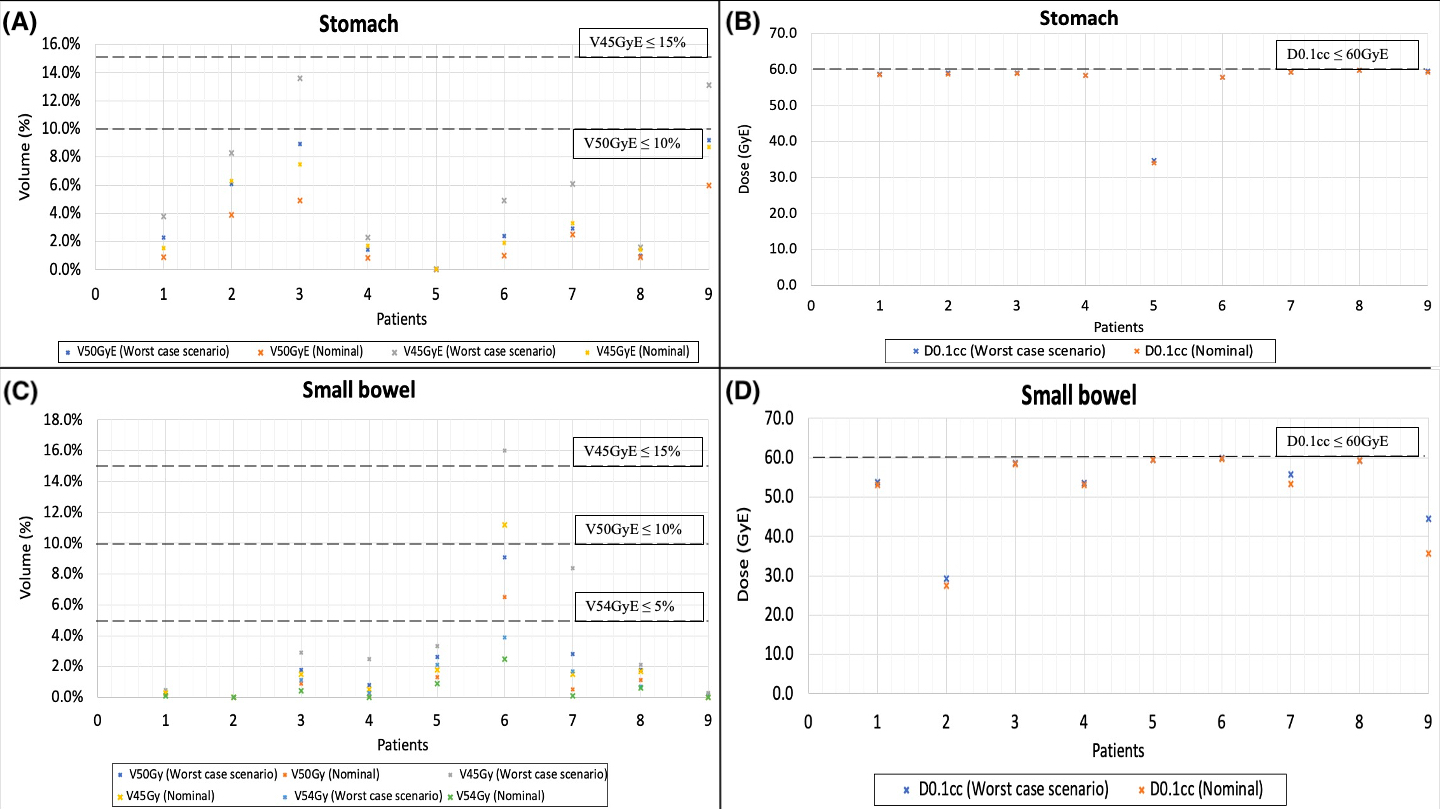

Supplement: Supplementary file 4 — Additional file 4: Figure 4. Stomach and small bowel dose with robustness analysis on the CTV. In this figure the nominal and worst-case scenario value for stomach and small bowel are shown. Dashed lines represent the dose constraints. (Abbreviations: DXcc: dose received by Xcc volume; VxGy: volume receiving X-Gy). [file 13014_2020_1592_MOESM4_ESM.jpg]

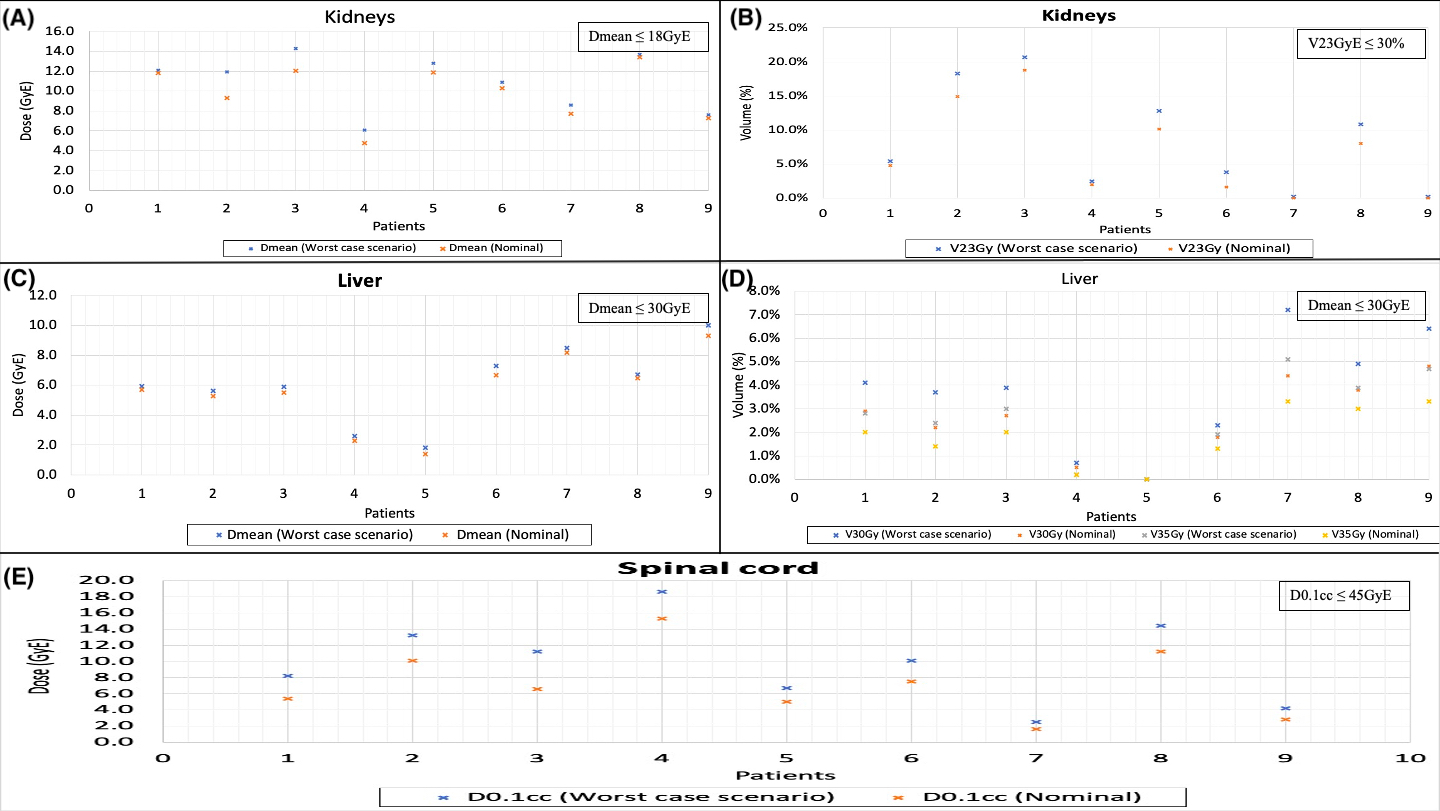

Supplement: Supplementary file 5 — Additional file 5: Figure 5. Kidneys, liver, and spinal cord dose with robustness analysis on the CTV at its worst iteration. In this figure the nominal and worst-case scenario value for Kidneys, liver, and spinal cord are shown. Dashed lines represent the dose constraints. (Abbreviations: Dmean: mean dose; DXcc: dose received by X cc volume; VxGy: volume receiving X-Gy). [file 13014_2020_1592_MOESM5_ESM.jpg]
