# Supplementary material for: Radiobiological model-based approach to determine the potential of dose-escalated robust intensity-modulated proton radiotherapy in reducing gastrointestinal toxicity in the treatment of locally advanced unresectable pancreatic cancer of the head
Source: Radiat Oncol. 2020 Jun 22;15:157. doi: 10.1186/s13014-020-01592-6 (PMC7310413; doi:10.1186/s13014-020-01592-6)
Supplement: Supplementary file 6 — Additional file 6. [file 13014_2020_1592_MOESM6_ESM.docx]

Details of IMRT and ro-IMPT planning, beam configuration, and optimization:

IMRT: The non-coplanar IMRT plan was made using Raystation (v6.2) TPS with collapsed-cone convolution-superposition (CCC)-based algorithm calculation (Raysearch Laboratories, USA) by setting a dose grid 0.2 x 0.2 x 0.2cm^3^. Dynamic multi-leaf collimator (MLC) delivery mode was used for the IMRT with a dose rate (DR) of 600 MU/min and a maximum leaf speed of 2.5cm/second. All the IMRT plan was delivered using 6 (10MV beam energy) beams, 4 co-planar beams (35-45^0^, 90^0^, 173-183^0^, and 310^0^) and 2 non-coplanar beams (gantry at 20^0^, 330^0^ with couch at 90^0^). The IMRT beam modeling was performed for the True beam (Varian Medical System, Inc., Palo Alto, CA). IMRT plans were optimized using Uniform, Max, Min, and EUD objective functions, dose constraints by trial and error method. An auxiliary ring structure of 5mm (RING ROI) was created 2cm away from the PTV to control the dose fall-off from the PTV. Also, for an adequate plan optimization, 40 iterations were performed.

IMPT: Non-coplanar CTV-based robust multifield optimization IMPT plan was made using Eclipse (v15.1) TPS (Varian Medical System, Inc., Palo Alto, CA) with a pencil beam dose algorithm and with a dose grid of 0.25 x 0.25 x 0.2 cm^3^ voxels. The beam modeling was performed for the proton therapy systems (Sumitomo heavy industry). All the ro-IMPT plan was delivered using 3 beams, 2 co-planar beams (135^0^ and 220^0^), and one non-coplanar beam (gantry at 270^0^ with couch at 5^0^) as shown in Figure 1.^19^ The optimization algorithm weighted all the beams. The ro-IMPT beam angles were adopted to reduce small bowel dose, simultaneously minimizing the degree of uncertainty for the organ motion and filling. The treatment planning calculations were based on beam data of Sumitomo scanning nozzle with a gaussian spot size with a nominal sigma of 6mm of air. Spot spacing was set to 0.5cm. The beam velocity limit for line scanning was 10mm/ms. A range shifter was inserted to ensure the application of spots close to the patient’s surface. The air gap was set to 2cm. The ro-IMPT plan optimization was performed by adjusting the dose, volume, and penalty of each objective for the target (CTV) and OARs. For a CTV-based ro-IMPT, as per current clinical practice at our institution, the setup uncertainties with the isocenter shift of ± 5mm in each orthogonal direction and range uncertainties of ± 3.5% of the beams nominal range were assumed in the optimization. In all IMRT and ro-IMPT treatment plans, the D_mean_ of CTV was normalized to the prescription dose.

Twelve worst-case dose distribution scenarios were computed based on the nominal treatment plan considering ± 5mm isocenter shift of the patient in each orthogonal direction and with a density error of ±3.5%, to confirm the robustness of the created ro-IMPT plan. The IMPT plan was defined as robust if the dose coverage in each of the 12 worst-case scenarios met at least the CTV dose constraints of V_95%_ ≥ 98% and D_0%_ ≤ 107% as shown in Additional Figure 2. The clinical objective in the worst-case scenarios were examined using either the voxel-wise minimum or maximum goals. The CTV [D95%, D0%] DVH metrics were calculated based on voxel-wise min distribution, and the DVH metrics in GI-OARs, liver, kidneys, and spinal cord were computed based on voxel-wise max distribution.
